# Endocytic Pathways and Recycling in Growing Pollen Tubes

**DOI:** 10.3390/plants2020211

**Published:** 2013-04-03

**Authors:** Elisabetta Onelli, Alessandra Moscatelli

**Affiliations:** Dipartimento di Bioscienze, Universita’ degli Studi di Milano Via Celoria 26, 20133 Milano, Italy; E-Mail: elisabetta.onelli@unimi.it

**Keywords:** pollen tube, polarized growth, clathrin-dependentendocytosis, clathrin-independentendocytosis, exocytosis, membrane recycling, actin cytoskeleton

## Abstract

Pollen tube growth is based on transport of secretory vesicles into the apical region where they fuse with a small area of the plasma membrane. The amount of secretion greatly exceeds the quantity of membrane required for growth. Mechanisms of membrane retrieval have recently been demonstrated and partially characterized using FM (Fei Mao) dyes or charged nanogold. Both these probes reveal that clathrin-dependent and -independent endocytosis occur in pollen tubes and are involved in distinct degradation pathways and membrane recycling. Exocytosis, internalization and sorting of PM proteins/lipids depend on the integrity of the actin cytoskeleton and are involved in actin filament organization. However, some kinds of endocytic and exocytic processes occurring in the central area of the tip still need to be characterized. Analysis of secretion dynamics and data derived from endocytosis highlight the complexity of events occurring in the tip region and suggest a new model of pollen tube growth.

## 1. Introduction

Pollen tubes are cell protrusions arising from pollen grains. They play a role in transporting and releasing sperm cells in the embryo sac for double fertilization. Pollen tubes follow a polarized growth model: their organelles are not uniformly distributed and vesicles accumulate in the apical region or clear zone (5–10 μM from the tip) forming an inverted cone-shaped domain. This area is succeeded by a subapical organelle-rich region, as well as nuclear and vacuolated zones [1]. During pollen tube growth, areas progressively distant from the tip become isolated by deposition of callose plugs [2,3,4].

For the last few years, the prevailing pollen tube growth model has assumed that the whole apex is the site of exocytosis. According to this model, Golgi-derived secretory vesicles fuse with the apical plasma membrane reversing outside cell wall material and providing new plasma membrane tracts for tube elongation [2,5]. The efficiency of tip growth is maintained by accumulation of secretory vesicles in the clear zone of the tip region. This cytoplasmic zonation is generated by actin-myosin-dependent reverse-fountain cytoplasmic streaming that conveys secretory vesicles to the tip region, where some fuse with tip plasma membrane and others are transported back again [6,7]. Light microscopy observations suggest that directed movements do not occur in the tip, as the motion of secretory vesicles in the clear zone is postulated to be governed by diffusion and advection [4,8,9]. This model is also supported by actin filament (AF) distribution pattern, which shows long longitudinally oriented AFs in the shank and fine networks of AFs in the apex, assumed to trap secretory vesicles in the clear zone [6,10]. Drugs affecting the integrity of AFs are known to block cytoplasmic streaming and to impair pollen tube growth. Low concentrations of latrunculins B (LatB) that do not inhibit cytoplasmic streaming were also found to affect tube elongation, suggesting that two distinct populations of AFs (long AF bundles in the shank, and fine AF network in the apex) act synergistically to convey secretory vesicles to the tip and to regulate exocytosis, respectively [7]. The observation of coated vesicles in subapical regions of the tube suggested, moreover, that endocytosis occurs in the shank in order to recycle an excess of secreted membrane [11]. Improved fixation methods [12] and studies of AF dynamics showing that AF behavior changes cyclically, in line with secretion and growth [13,14,15], and new insights derived from studies of membrane trafficking suggest complex composition and trafficking of vesicles in the apex and in subapical regions. These new data are discussed in order to contribute to an integrated tip-growth model.

Recent studies on endocytosis in pollen tubes with fluorescent probes, such as FM 4-64 or FM 1-43 and charged nanogold, suggest that V-shaped accumulation of vesicles in the clear zone include secretory vesicles and newly internalized endocytic vesicles [8,16,17]. The polarized growth of pollen tubes is supported by a delicate equilibrium between exocytic and endocytic pathways. In the cortical region of the pollen tube, organelles and secretory vesicles move towards the apex. Near the apical region, some vesicles are captured and maintained in the clear zone, while other vesicles and organelles are redirected towards the central pollen cytoplasm [4,18,19]. In this way, secretory vesicles derived from Golgi apparatus are directed to specific domains of the apical plasma membrane. Endocytic vesicles are the smallest vesicles in pollen tubes and have a role in retrieving and recycling excess secreted plasma membrane [11,20], thus regulating internal membrane economy [18,21]. This new data has contributed significantly to formulation of a new model of pollen tube growth.

## 2. Endocytic Pathways

The study of plant endocytosis is a relatively new field. Until the early 1900s, it was generally thought that endocytosis was energetically unfavorable in walled cells because of the presence of the turgor pressure [22]. Clathrin-dependent and clathrin-independent internalization pathways have since been described in plants. During clathrin-dependent endocytosis, internalized plasma membrane is delivered to the trans-Golgi network/early endosomes (TGN/EE) [23,24,25]. The TGN appeared to be a highly dynamic and independent organelle, only temporarily associated with Golgi stacks. Some evidence suggested that endocytosis and exocytosis intersected in this organelle. In fact, inhibition of an isoform of vacuolar H^+^-ATPase by concanamycin A causes accumulation of both endocytic and exocytic vesicles in the TGN [23]. Inhibition of exocytosis was recently found in the ECHIDNA mutant (*ech*) of *Arabidopsis thaliana* due to misallocation of certain proteins that localize to or traffic via TGN/EEs [26]. However, endocytosis was not affected, suggesting that exo- and endocytosis could be located in different TGN domains [26]. After internalization, endocytic vesicles destined for degradation or recycling to the plasma membrane could therefore be sorted through distinct TGN subdomains or through a putative recycling endosome. The degradation pathway involves additional organelles, such as multivesicular bodies/late endosomes (MVBs) and lytic vacuoles. Cargoes destined for degradation are trapped in the internal vesicle system of MVBs and delivering of plasma membrane proteins/lipids to vacuoles requires previous ubiquitination, which is the signal for ESCRT-dependent sorting to the degradation pathway [27]. Four different ESCRT-complexes are involved in vacuolar degradation: ESCRT 0, I and II recognize and concentrate ubiquitinated cargoes within EEs, preventing their recycling to the plasma membrane. Subsequently, ESCRT III and ESCRT-associated proteins play a role in EE membrane invagination, determining the inner morphology of MVBs [28]. ESCRT-mediated sorting of cargo destined for degradation therefore occurs in TGN/EE and it is hypothesized that MVBs originate from the maturation of specific TGN/EE domains [29]. 

Charged nanogold probes for electron microscopy identified a compartment characterized by interconnected tubules and cisternae, identified as an EE, in tobacco protoplasts [30]. The roundish area of this compartment showed an internal membrane system similar to that of MVBs, suggesting that MVBs could be derived from a specific domain of this early compartment. In Arabidopsis and BY2 cultured cells, the distribution of ESCRT complexes and annexins in TGN/EE and MVBs suggests that MVBs are derived from maturation of TGN and that trafficking to vacuoles does not involve shuttle vesicles [29]. In fact, ultrastructural observations showed that MVBs directly fuse with vacuoles [29,30], in which lipases and proteases degrade intraluminal vesicles released by fusion of the MVB-delimiting membrane with the tonoplast. In a recent paper, mechanisms allowing cargo delivery to vacuoles and recycling of vacuolar-sorting receptors from late compartments was hypothesized [31]. The authors proposed the presence of an intermediate compartment, which matures from MVBs and is named *late prevacuolar compartment* (LPVC), between MVBs and the vacuole. Although the LPVC resembles MVBs morphologically, lipid and protein composition of the organelle-delimiting membrane is modified during the maturation process. Vacuolar-sorting receptors are depleted from the external membrane of MVBs by selective retrieval involving the retromer complex [32]. Internal vesicles are not affected by this process and are destined for degradation. During this membrane recycling process, LPVC acquires the competence to fuse with the vacuole [31].

**Figure 1:**
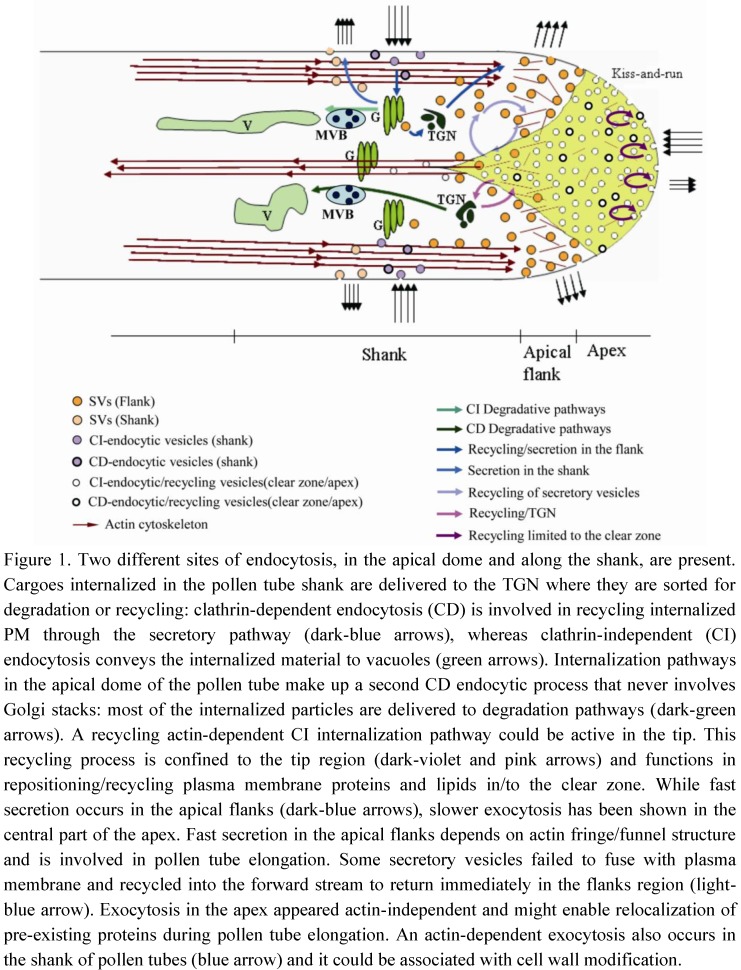
Model of exocytic/endocytic pathways contributing to pollen tube growth.

^−^Ng showed the morphology of the internalization pathway occurring in the tip region. ^−^Ng is internalized in the apical dome of the pollen tube and is never observed in Golgi stacks. Most internalized particles are delivered to degradation pathways through the TGN. Moreover, pulse-chase experiments clearly showed a recycling pathway confined to the tip region (Figure 1). This pathway could not involve TGN/EE function [16], since secretory carrier membrane proteins (SCAMPs), which characterize TGN/EE in tobacco BY-2 cells [24], were mostly localized in small apical vesicles and secondarily in TGN and vacuoles in lily pollen tubes [34]. While this protein characterizes EEs, it may be hypothesized that an early sorting station, before the TGN, could exist in the apex and enable faster recycling of internalized plasma membrane limited to the tip. This tip-limited recycling process may not be involved in pollen tube growth but could take part in redistribution of protein complexes or lipids between different membrane domains in order to maintain functional specialization in the apical plasma membrane. These protein complexes may include factors involved in ionic regulation, such as Ca^2+^ channels, H^+^ ATPases or K^+^ and Cl^−^ transporters [35,36], or in signal transduction, such as Rac/Rop GTPases (Figure 2). These proteins are involved in pollen tube–pistil interactions and are key regulators of tip growth [37].

In tobacco pollen tubes, negatively (^−^Ng) and positively (^+^Ng) charged nanogold and the lipophilic dye FM4-64 showed two different sites of endocytosis: in the apical dome and along the shank (Figure 1). Time-lapse experiments using FM4-64 showed that internalization in the apex was significantly lower than in the shank, suggesting for the first time that not only exocytosis, but also endocytosis, occur in the tip region and that the clear zone could be the site of accumulation of transport vesicles and not just secretory vesicles [16]. The combination of FRAP (fluorescence recovery after photobleaching) and STICS (spatiotemporal image correlation spectroscopy) analysis allowed to describe and measure the movement of apical vesicles [8]. FRAP experiments with the fluorescent dye FM1-43 in lily pollen tube showed that, when the probe was removed from the medium, fluorescent recovery in the extreme apex was delayed in time if compared with the lateral areas of the apex, suggesting that endocytosis occurs in this central zone [8]. Mathematical modeling on membrane flow and cytosis in the clear zone of pollen tube showed that vesicles in this area consist mostly of endocytic vesicles [9,33]. Moreover, the theoretical model predicted a very good spatial separation between endocytosis and exocytosis, which originates the bidirectional flow of vesicles and maintains cell polarity [33]. Different dyes, namely FM4-64 and FM1-43, showed that endocytosis in pollen tubes involves formation of small vesicles in the clear zone and larger vesicles in the shank: the former enter the endosomal retrograde trafficking stream and the latter could be clathrin-coated endocytic vesicles [17]. Experiments of endocytosis dissection with charged nanogold confirmed the presence of distinct endocytic pathways in pollen tubes. ^+^Ng was internalized in the pollen tube shank and delivered to the TGN where it was sorted for degradation or recycling (Figure 1). As in somatic cells, also in pollen tubes, TGN/EE appears to be a first sorting station for MVBs and vacuoles [16]. Moreover, most vesicles internalized in the shank are recycled to the apex of the pollen tube through the Golgi apparatus. The probe was observed in cis and medial Golgi stacks and was recycled to the plasma membrane by exocytosis. Thus, the plasma membrane internalized in the pollen tube shank is largely reused for secretion and pollen tube growth (Figure 1) [8,16].

**Figure 2:**
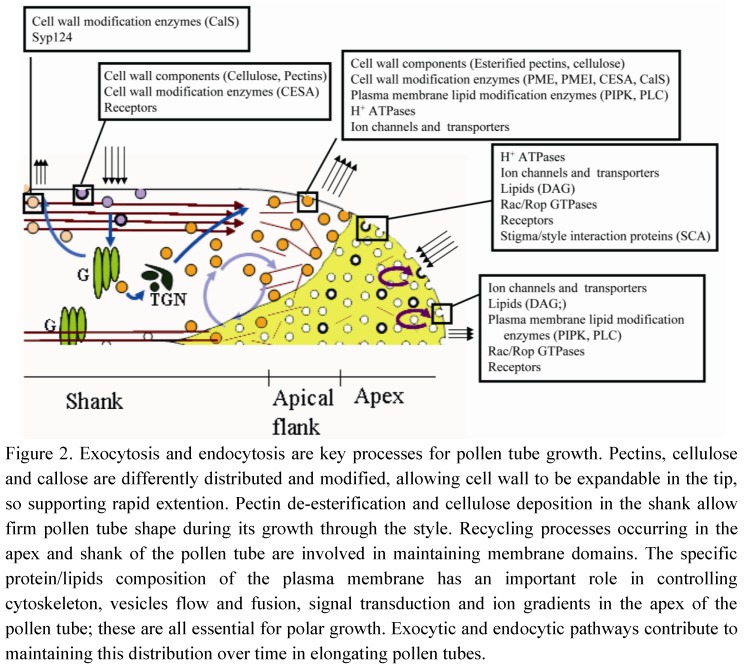
Exocytic/endocytic pathways of molecules involved in pollen tube growth.

## 3. Exocytosis/Recycling

Rapid pollen tube growth is supported by the fusion of Golgi-derived secretory vesicles to a restricted area of the tip that supplies new segments of plasma membrane and new cell wall components (Figure 1). Different populations of vesicles have been described in pollen tubes: the smallest accumulate in the central portion of the apex, while the largest are observed in the distal region along the shank [17]. These vesicles could represent tip-internalized and clathrin-dependent endocytic vesicles, respectively. Vesicles with intermediate diameter occurring in an area adjacent to the tip, 3–10 μm distal to the apex, are exocytic vesicles [17]. The distribution of secretory vesicles and the specific site of secretion are correlated with the distribution of actin filaments (AFs) [9,12]. The actin cytoskeleton plays a central role in pollen tube growth [6,14,38] and shows different structural organization in the shank and tip [12,39]. Thick longitudinally oriented actin cables in the shank reach the inverted cone region and contribute to the reverse fountain cytoplasmic streaming pattern. Actin bundles never extend to the apical clear zone, where diffuse staining suggests the presence of single and more dynamic AFs. In the apical flanks (2–5 μm from the apical plasma membrane; [36]) a dynamic network of short actin cables forms different arrays, referred to as ring, funnel, basket, mesh and fringe [7,12,40,41,42]. These actin structures define the region where secretory vesicles preferentially fuse with the plasma membrane and alternate in the tip during pulsed growth [43,44]. Secretory vesicles are delivered to a position that corresponds exactly to the proximal end of the actin fringe. Vesicles are carried in very close proximity to the plasma membrane increasing the chance of secretory vesicles to release their content in this area [8,45]. Moreover, modeling of vesicle dynamics showed that actin polymerization drives the advance of apical fringe and constrains the movement of vesicles in the inverted cone: the orientation of the microfilaments determine the direction in which vesicles are delivered or removed from this area [9,45]. Movement of vesicles in the cone-shaped region was not actin-myosin dependent [9]. Thus, in the apical flanks, actin filaments deliver secretory vesicles to the plasma membrane while excess secreted plasma membrane is retrieved by endocytosis (Figure 1). 

FRAP experiments in different regions of the apex of tobacco pollen tubes stained with FM4-64 led to the hypothesis that exocytosis actually occurs in both the apex and apical flanks (Figure 1). While fast secretion occurs in the apical flanks, slower exocytosis has been shown in the central part of the apex [39] (up to 2 µm from the apical plasma membrane), suggesting that secretion in different domains of the clear zone relies on distinct mechanisms and probably accomplishes different functions. Fast secretion in the apical flanks depends on actin fringe/funnel structure, since low concentrations of LatB affect the speed of fluorescence recovery in the apical flanks and not in the apex [39]. 

In Arabidopsis, a subunit of the putative exocyst complex (SEC8) seems to be involved in pollen tube germination and growth [46]. Exocyst is a tethering protein complex that targets secretory vesicles to specific sites of the plasma membrane. In pollen tubes, SEC8 defines the sites for localized exocytosis during early stages of polarized growth in pollen grains and is also involved in the subsequent elongation. In maize, exocyst components are required for normal exocytosis in other tip-growing cells, such as those of root hairs [47]. It is therefore possible to speculate that a putative exocyst complex could also be involved in delivering secretory vesicles from Golgi apparatus to pollen apical flanks, where fast exocytosis occurs.

An actin-independent recycling process has also been hypothesized in the tip region (Figure 1). Studies on Arabidopsis pollen tubes have shown that delivery of proteins to specific areas of the apical plasma membrane does not involve targeted secretion and might enable relocalization of pre-existing proteins during pollen tube elongation [48]. It has been shown that proteins/lipids repositioning between different plasma membrane domains of the tip (apex and apical flanks) require fine regulation between exo- and endocytosis (Figure 2). Among these proteins, Rac/Rop-GTPases are localized in a restricted area of the apical plasma membrane and are key regulators of polar cell expansion [43,49,50,51,52]. Rac/Rop-GTPases are considered to be intracellular mediators that translate signals from pistil-transmitting tissue into specific growth patterns, and that guide pollen tubes to the embryo sac [26]. In addition, Rac/Rop-GTPases regulate AF dynamics by acting on formins, which are actin-nucleating proteins [53,54]. Formins (FH) are known to regulate actin-related processes and coordinate actin and microtubule MT function in HeLa cells. FH2 mediates the action of a protein (mDia1) that orientates MTs, enabling them to align with actin bundles [55]. Since mDia1 interacts in turn with members of the Rho family, the authors proposed that these proteins could connect the GTPases functionally to the actin cytoskeleton [55]. Close association between MTs and AFs has been observed in the subapical regions of pollen tubes after rapid freeze fixation and substitution [56,57] and MT organization seems to depend on correct organization of the actin cytoskeleton. Low concentrations of LatB revealed dramatic reorganization of the MT cytoskeleton, leading to the formation of MT bundles, often organized in rings, in the apex and shank of tobacco pollen tubes [58].

In pollen tubes, FH3 and FH1 stimulate actin cable formation and actin assembly along the shank, where AFs are responsible for cytoplasmic streaming. Overexpression of these proteins induces cell membrane deformation, suggesting a role in polar growth [59,60]. Since pollen tube elongation is closely coupled with actin polymerization, it has been suggested that properly regulated membrane-associated actin polymerization induced by formin could contribute to protrusive growth at the tip. A differently localized formin, FH5, plays a role in assembling actin structures just behind the apical flanks [61]. In Arabidopsis, FH5 is associated with the apical membrane of pollen tubes and contributes to the organization of subapical actin, giving rise to the mesh-like structure in the apical flanks. These actin cables provide the tracks for vesicles destined for fast exocytosis, as well as for vesicles/organelles that reverse their motion and are transported back into the central cytoplasm [9,61]. RNAi experiments or antisense transgenes against Nt-FH5 have shown that actin polymerization stimulated by FH5 is important for maintaining the polarized distribution of vesicles and organelles [61].

To maintain different domains in the apical pollen tube plasma membrane, Rac/Rop-GTPases interact with factors other than formins, such as phosphatidylinositol monophosphate kinase (PIP-kinase). This kinase synthesizes phosphatidylinositol 4,5-biphosphate (PIP_2_), a possible effector of Rac/Rop in pollen tubes, and may play a central role in actin dynamics, vesicle trafficking and ion transport [62]. In animals, PIP_2_ synthesis allows Rac/Rop-GTPases to control secretion regulating cytoskeletal organization and exocytic membrane trafficking [63,64]. In pollen tubes, PIP_2_ is localized in the tip and seems to be directly involved in controlling actin-mediated targeted secretion, modifying membrane lipid composition and recruiting proteins involved in membrane fusion [43]. PIP-kinases and phospholipase C (PLC) help maintain distinct domains, involved in exocytosis and endocytosis, in the tip of growing pollen tubes. Two isoforms of these enzymes (PIP5K4 and PLC3) show different but complementary distribution: PIP5K4 localizes in the central part of the apex, whereas PLC3 is observed at the edges of the apex/apical flanks [65,66] and this distribution appears to be constant in time in elongating pollen tubes (Figure 2). This distribution keeps PIP_2_ confined to the central part of the tip: inhibition of PLC activity results in lateral spreading of these lipids, inhibiting polarized growth [65]. PIP_2_ is therefore generated in the apex by PIP-kinase activity, and is hydrolyzed by PLC3 in the apical flanks, where PLC distribution overlaps with that of its substrate. In petunia, moreover, PLC1 showed a similar localization and helps restrict growth to the apex by regulating the distribution of PIP_2_ [67]. Hydrolysis of PIP_2_ results in the formation of 1,4,5-triphosphate (IP_3_) and diacyl glycerol (DAG), which are both involved in controlling polarized growth. DAG is endocytosed at the edges of the apex and recycled to the central part of the apical plasma membrane (Figure 2). Maintenance of asymmetrical distribution of these lipids is due to coordination of exocytic/endocytic processes (Figure 2). Although the apical plasma membrane domains are enriched in PIP_2_ and DAG, which seem to be essential for secretion and pollen tube apical elongation, the exact nature of their role remains to be determined. PIP5K4 and PIP5K5, isoforms of Arabidopsis PIP-kinases, seem to play an important role in the secretion of pectins in the apical flanks, regulating the plasticity of the apical cell wall [68]. A second product of PLC activity is IP_3_ that plays a crucial role in establishing the Ca^2+^ gradient and in regulating Ca^2+^ signaling [69]. Thus, close regulation of IP_3_ and PIP_2_, modulating homeostasis and secretion of Ca^2+^, is necessary for proper pollen tube growth [70]. An interaction between PIP_2_ and Rac/Rop signaling was recently demonstrated [71]. Two isoforms of A phosphatidylinositol 5-kinase are exclusively expressed in pollen tubes and also catalyze PIP_2_ formation in the apex. The distribution of these kinases partially overlaps with the B types already described, since they have been observed in the apical flanks and shank of pollen tubes [71]. The different distribution with respect to B type could be due to interaction with specific partner lipids/proteins, which recruit these enzymes to different functional domains [71]. The activity of A kinases is also related to actin cytoskeletal integrity in the tip, since their overexpression induces the formation of actin cables or actin aggregates extending into the clear zone and disappearance of the actin fringe. It has been hypothesized that PIP_2_, derived from catalytic activity of type A kinases, affects Nt-Rac5 functions, stimulating membrane association and activation of this Rac/Rop GTPase [72]. Tip localization of PIP_2_ and Nt-Rac5 is also finely regulated by recycling processes that reposition different enzymes and Rac/Rop factors in the apex of growing pollen tubes [71,73] (Figure 2). Slower exocytosis in the central area of the apex could be involved in repositioning signaling proteins, such as Rac/Rop GTPases, PLCs and lipids, in the clear zone, in the meantime recycling putative receptors to the plasma membrane for internalization by clathrin-dependent, receptor-mediated endocytosis in the apex (see below; Figure 2). The different dynamics of this actin-independent exocytic pathway suggest different regulation. It was generally thought that apical vesicles residing in the clear zone are only subject to Brownian motion [10,74] and it is still unclear whether the cytoskeleton plays a role in vesicle recycling in this zone.

FRAP experiments performed in the shank of pollen tubes showed that exocytosis also occurs in this area (Figure 1) [8,39,75] and LatB experiments demonstrated that the process is actin-dependent. In a recent report, expression of a pollen-specific syntaxin SYP124, required for docking and fusion of secretory vesicles, was observed not only in a limited membrane domain in the apical flanks, as expected, but also in the shanks of pollen tubes (Figure 1). This exocytic process did not seem to be associated with pollen tube growth and will be further characterized [76]. Secretion in the shank could be associated with cell wall modification (Figure 2). Pectins are secreted in their esterified forms in the apical region and are subsequently de-esterified by the enzyme pectin methyl esterase that is co-secreted with esterified pectins [2,77,78]. Pectin de-esterification, callose deposition and regulation of cellulose repositioning occur between the apical flanks and shank areas of cell walls [79] (Figure 2). Methylesterified pectins characterized tip cell wall which remains expandable, thus supporting rapid growth. Otherwise, pectin de-esterification allowed the formation of Ca^2+^ bridges among galacturonic acid residues causing cell wall rigidification that contributes to maintain pollen tube shape during its growth through the style [80]. PME and its inhibitors (pectin methylesterase inhibitor, PMEI) are secreted in the flanks of pollen tube and contribute to maintain cell wall plasticity: endocytosis in the shank allows for the internalizing of PMEI. Thus, PME can modify pectins in this area while in the tip PMEI accumulates and inhibits de-esterification of cell wall components [81,82]. This different distribution of pectins is important for the geometry of the elongating tube [80]. Cellulose and callose are also present several microns behind the tip [83]. Cellulose synthase is secreted in the apical flanks and deposition of cellulose begins immediately. As the pollen tube grows, cellulose could be progressively removed from the shank by endocytosis and relocalized in the apical flanks [79]. Callose synthase (CalS) also accumulates in the distal pollen tube plasma membrane and is involved in cell wall modification and formation of callose plugs [84]. In *Nicotiana alata*, a protein involved in callose deposition (glucan synthase-like 1; NaGSL1) is secreted in the pollen tube shank, where callose synthesis begins [85]. Immunogold labeling experiments have shown that inactive CalS localizes in ER, Golgi bodies and vesicles lying under the plasma membrane. The active enzyme is incorporated into the plasma membrane and contributes to callose deposition in shank cell walls [85]. The delivery and accumulation of CalS in the distal region depends on AFs, while MTs appear to be significantly involved in the distribution and maintenance of distal CalS [84].

## 4. Clathrin-Dependent and Clathrin-Independent Endocytosis

In plant cells, clathrin-dependent endocytosis has been well characterized and probably accounts for most endocytic processes into the cell, whereas clathrin-independent endocytosis seems to play a minor role [86]. However, there is some evidence of both clathrin-dependent and clathrin-independent endocytosis in plant cells [30,87,88]. In animal cells, clathrin-independent endocytosis may occur in lipid raft domains [89]. Lipid-dependent endocytosis follows the pattern of internalization described for clathrin-dependent endocytosis and probably also involves the endoplasmic reticulum (ER) [90]. In plants, raft domains show the same general model as in animal membrane studies but with phytosterols as central structural elements [91]. Proteins associated with these lipid platforms are involved in signaling, trafficking and cell wall metabolism [92,93,94]. There is little evidence of lipid raft involvement in endocytosis in plants. In a recent paper, it was shown that a protein, flotillin 1 (Flot1), identified as a component of lipid rafts in plants, participates in clathrin-independent endocytosis [95]. In animals, flotillins induce membrane curvature and plasma membrane invagination during internalization processes [96]. In Arabidopsis root cells, Flot1 partially colocalizes with endocytic marker FM4-64 and is observed in plasma membrane invaginations leading to large vesicle formation, TGN, endosomes, and ER [95]. Membrane domains showing Flot1 are distinct from plasma membrane domains coated in clathrins by virtue of their localization and dynamic behavior. Endocytic processes depending on Flot1 seem to require the integrity of sterol-rich membrane microdomains and cytoskeleton: AF and MT depolymerizing drugs have shown that MTs are required for efficient distribution/dynamics of microdomains containing Flot1. This data suggests that clathrin-independent endocytosis involving Flot1 and probably sterol-rich membrane microdomains occurs in *Arabidopsis* root cells [95].

In pollen tubes, both clathrin-dependent and clathrin-independent endocytic processes are involved in recycling different PM proteins/lipids in the apex and in regulating pollen tube growth [16]. ^+^Ng and ^−^Ng combined with an inhibitor of clathrin-dependent endocytosis (ikarugamycin, Ika) have shown that clathrin-dependent and clathrin-independent endocytosis occur in different regions of pollen tubes and are involved in different internalization pathways [16]. Ika showed clathrin-dependent and clathrin-independent internalization of ^+^Ng in the shank: clathrin-dependent endocytosis is involved in recycling internalized plasma membrane through the secretory pathway, whereas clathrin-independent endocytosis conveys the internalized material to vacuoles (Figure 1). The dimension of coated endocytic vesicles 10–15 μm from the tip suggests that clathrin-dependent endocytosis occurs in this pollen tube area [11,17]. ^−^Ng detects a second clathrin-dependent endocytic process occurring at the tip, whereby internalized material is sorted to vacuoles without intersecting the secretory pathway (Figure 1). ^−^Ng has been observed in TGN but not in Golgi stacks, suggesting another degradation pathway depending on the formation of clathrin-coated vesicles. This pathway could be involved in internalizing factors that mediate pollen tube-pistil interactions (Figure 2). Stigma/style Cys-rich adhesin (SCA) is a protein involved in pollen tube adhesion, a mechanism guiding pollen tubes toward the ovary [97]. This protein is internalized in the pollen tube tip by clathrin-dependent endocytosis and follows the pattern observed for ^−^Ng, reaching MVBs and vacuoles but bypassing Golgi apparatus [98]. Thus, clathrin-dependent and clathrin-independent degradation pathways coexist and are localized in different areas of the pollen tube plasma membrane.

Time-lapse experiments using FM4-64, with or without Ika, show only partially reduced internalization in the tip and shank. Pulse-chase experiments using ^−^Ng suggest that some vesicles recycle in the apical region without leaving the clear zone. Although Ika treatment severely impairs uptake of the probe destined for vacuoles, it is not clear whether the endocytic pathway involved in relocalizing apical plasma membrane proteins/lipids is also affected by the clathrin-dependent inhibitor [16].

In animals, PIP_2_ is required at synapses for exo- and endocytic cycling of presynaptic vesicles and secretory granules and plays an important role in regulating clathrin-mediated endocytosis. PIP_2_ appears essential for defining and stabilizing endocytic sites and is involved in recruitment of adaptor protein AP-2 to the plasma membrane for coated-vesicle formation [64]. In Arabidopsis, salt stress induces PIP_2_ to associate with clathrin-coated vesicles, suggesting that this lipid is also involved in endocytic processes, as demonstrated in synapses [99]. Moreover, clathrin-dependent endocytosis in pollen tubes has been shown to depend on a proper balance between phosphatidylinositol 4-monophosphate (PI4P) and PIP_2_ [100]. PIP5K6, a kinase for PIP_2_ formation, is localized in the subapical region of growing pollen tubes. Overexpression and silencing of PIP5K6 show that PIP2 is important for the activation of early stages of clathrin-dependent endocytosis, which promote the formation and invagination of clathrin-coated pits in the apical plasma membrane, while PI4P is involved in later stages of clathrin-coated vesicle formation in tobacco pollen tubes [100]. Although PIP_2_ promotes the formation of coated pits in animals and yeast by recruiting AP-2 complex, at the moment there is no evidence of any interaction of this lipid with the AP-complex in plants. In addition, since PIP_2_ seems to be involved in regulating actin cytoskeletal dynamics [62,67], the interaction between PIP_2_ and actin could be a factor that affects clathrin-coated vesicle formation. ^+^Ng and ^−^Ng used as endocytic probes, with actin dynamics perturbed by low concentrations of LatB, have shown that clathrin-dependent and CI endocytosis in the shank of pollen tubes are both affected by the drug, whereas the clathrin-dependent degradation pathway occurring in the tip region is not significantly affected by LatB [39]. However, quantitative analysis of ^−^Ng particles in the clear zone show a decrease in the number of vesicles stained by the probe, suggesting that internalization in the tip is also partially impaired by LatB.

An actin-dependent clathrin-independent internalization pathway could therefore be active in the tip of pollen tubes (Figure 1), with the function of repositioning/recycling plasma membrane proteins/lipids in/to the clear zone. The hypothesis that some vesicles in the clear zone do not enter in cytoplasmic streaming but remain in the tip, eventually re-fusing with the plasma membrane, is sustained by studies carried out in lily and *Petunia inflata* [8,101]. A pollen-specific SNARE, longin PiVAMP/726, seems to play a role in endocytic recycling occurring at the tip, regulating direct fusion of newly internalized endocytic vesicles with exocytic vesicles derived from the TGN [101]. Moreover, the combination of FRAP and STICS analysis in lily pollen tube showed that many secretory vesicles, once arrived in the flanks, detached from actin filaments and failed to fuse with plasma membrane. Therefore, they are recycled by the forward stream to the flanks region [8] (Figure 1; light-blue arrows). Alternatively, it has been proposed that the apex could be a site of “kiss-and-run” endocytosis [8]. This type of endocytosis has been described in synapses as a clathrin-independent internalization process [102]. In addition, bulk endocytosis is involved in fast, apical recycling mechanism in synapses [102]. Although there is still no evidence of such processes in plants, they may occur in cells where rapid and extensive retrieval and recycling of plasma membrane is necessary.

There is evidence that a kinesin-like protein is associated with vesicles in the tip region and that stained vesicles are associated with short MT strands in the clear zone [103,104,105]. Although kinesin is known to play a role in driving the movement of organelles along MTs in *in vitro* assays, the identity of these organelles is still unknown [106,107]. Clathrin-dependent and clathrin-independent degradation pathways could therefore be regulated by different mechanisms involving MTs instead of AFs in the tip region and the putative microtubular motor proteins observed in this area could play a role in the apex localized vesicle trafficking.

## 5. Conclusions

Data reported for endocytosis and membrane recycling in pollen tubes suggest that endocytic pathways as clathrin-independent endocytosis, although not yet investigated, could contribute in understanding the fine equilibrium between exo- and endocytosis in polar growth. Moreover, studies carried out using LatB revealed actin-independent movements and suggested that MTs could cooperate with AFs in mediate vesicle movements in the apical region of the angiosperm pollen tubes.
